# Psychosocial factors affecting dietary habits of university students: A cross-sectional study

**DOI:** 10.1016/j.heliyon.2022.e09768

**Published:** 2022-06-22

**Authors:** Leila Cheikh Ismail, Tareq M. Osaili, Maysm N. Mohamad, Mona Hashim, Lily Stojanovska, Rameez Al Daour, Dalal Nader, Hanoof Alrayis, Nouf Sultan Alzaabi, Lojain Elbarag, Shaikha Binkhadim, Amjad H. Jarrar, Ayesha S. Al Dhaheri, Hayder Hasan

**Affiliations:** a Department of Clinical Nutrition and Dietetics, College of Health Sciences, University of Sharjah, Sharjah 27272, United Arab Emirates; b Nuffield Department of Women's & Reproductive Health, University of Oxford, Oxford OX1 2JD UK; c Department of Nutrition and Food Technology, Faculty of Agriculture, Jordan University of Science and Technology, Irbid 22110, Jordan; d Department of Nutrition and Health, College of Medicine and Health Sciences, United Arab Emirates University, Al Ain 15551, United Arab Emirates; e Nutrition and Dietetics Program, School of Health Sciences, Universiti Sains Malaysia, Kubang Kerian 16150, Kelantan, Malaysia; f Institute for Health and Sport, Victoria University, Melbourne 14428, Australia

**Keywords:** Body mass index, Eating habits, University students, Psychosocial factors

## Abstract

**Background:**

University student transition from living at home to more independent living which might influence their eating habits. This study aims to assess the effect of psychosocial factors on eating habits among university students.

**Methods:**

A cross-sectional study was conducted among 529 students at the University of Sharjah. Participants completed a self-administered questionnaire on dietary habits, social, and psychological factors. The height and weight were also measured.

**Results:**

More than one-third of participants were classified as overweight or obese (37.6%) and 39.1% reported not engaging in regular physical activity. Less than half of the participants consumed breakfast daily (45.4%) and 83.2% consumed less than two liters of water per day. Only 28.7% and 34.0% of participants consumed fruits and vegetables daily, respectively. Almost 80% of participants reported eating when they were bored, 83.7% ate when feeling happy, and 56.5% ate when they were sad. Eating habits score was significantly lower among unmarried participants (p = 0.03), those not living with their family (p < 0.001), smokers (p = 0.001), those not engaging in regular physical activity (p < 0.001), and those who reported eating uncontrollably (p = 0.007).

**Conclusions:**

Psychosocial factors were important indicators of dietary habits among students. Implementing nutrition education campaigns and health-related courses at the university are recommended.

## 1 Introduction

The United Arab Emirates (UAE) has experienced rapid advancement in the socioeconomic domains over the past 49 years. The gross domestic product have increased from 14.721 Billion in 1975 to 358.869 Billion in 2020 [1]. This progress has been accompanied by a nutritional transition in dietary habits from traditional to westernized types of foods [2, 3]. Additionally, other changes such as the adoption of energy-dense fast foods, eating away from home, frequent snacking, low intake of fruits and vegetables, high consumption of soft and energy drinks, increased rate of tobacco smoking, along with sedentary lifestyle habits have contributed to the growing rates of metabolic disorders among the Emirati population [4, 5, 6, 7, 8]. Unhealthy lifestyle and eating behaviors along with physical inactivity were also recently reported among UAE residents during the COVID-19 pandemic [9, 10]. Similarly, studies among university students in the UAE indicated a high prevalence of type 2 diabetes mellitus, hypertension, overweight, and obesity due to the above-stated reasons [11, 12, 13, 14]**.**

University years are a critical transition stage for students from adolescence to becoming independent adults. Students may move out from the family house and take complete responsibility for their lifestyle including their dietary habits [14]. Thus, they might adopt undesirable dietary and lifestyle behaviors, that could persist into their adult life.

The literature has reported inadequate nutritional knowledge related to dietary recommendations, sources of nutrients, and diet-disease relationships among university students in the UAE [13, 15]. Moreover, a study of female university students in the UAE reported poor eating habits such as high frequency of fast food consumption, tendency to skip breakfast, failing to meet the recommended intakes of whole grains, fruits, and vegetables, and physical inactivity [16]. Another study indicated that university students in the UAE tend to purchase foods and drinks from the vending machines due to easy access, lack of time, and peer influence [17], even though neither the snacks nor beverages offered in vending machines were nutrient-dense [18]. Additionally, students in the UAE reported facing academic-related stress, psychosocial stressors, emotional challenges, and socioeconomic concerns [19]. Stress among university students has been linked to poor dietary choices, increased body mass index (BMI), smoking, and higher food intake [20, 21, 22, 23]. Moreover, previous studies suggested multiple psychological factors related to overeating and emotional eating among university students such as perceived stress, depression, boredom, and anxiety [24, 25].

Considering the social and psychological influences accompanying the transition to university, we hypothesized that university students might exhibit poor eating habits and primary risk factors for chronic diseases. There are limited studies from the UAE on the dietary behaviors of university students. Thus, the present study aimed to assess the effect of social and psychological factors on eating habits among students at the University of Sharjah (UOS), UAE.

## 2 Materials and methods

### 2.1 Study design and subjects

This cross-sectional study was conducted among students at the UOS during the spring semester, 2018. A proportional quota sampling method was used to recruit students from medical and non-medical campuses. This is a non-probability sampling method that requires the groups being studied (medical versus non-medical students) to be proportional to the population being studied. Subjects were selected by convenience until the specified number of subjects for each subgroup was reached. A sample of 529 students (49.5% medical, and 50.5% non-medical) participated in the study. Each participant was given an information sheet explaining the objective and nature of the study. Participation was voluntary; students who agreed to take part in the study were asked to sign a written consent form according to the Helsinki declaration (Supplementary File S1). Participants were encouraged to ask questions if they needed clarification. All data were collected anonymously with no indication of any personal information and participants were not rewarded. Participants were allowed to withdraw from the study at any given time. The study protocol was approved by the Research Ethics Committee at the UOS (REC-18-03-08-01-S).

### 2.2 Data collection

Consented participants were asked to complete a multicomponent, self-administrated survey in the English language. The questionnaire contained questions on dietary habits and psychological factors affecting food choices. A researcher from the College of Health Sciences at the UOS developed the draft survey. The survey questions on dietary habits were developed based on previously published studies on university students [26, 27]. The questions on psychological factors were adapted from the validated Compulsive Eating Scale (CES) [28]. The survey was then reviewed by the research team. Twenty students from UOS were recruited through convenience to pilot test the survey. Following the pilot testing, only minor modifications in wording were made to the survey. Data from the pilot testing was not included in the final analysis. The internal consistency of the Compulsive Eating Scale (CES) was assessed using Cronbach's alpha test and it was determined at 0.82.

The questionnaire consisted of three sections. The first section included questions about sociodemographic data, such as age, gender, nationality, living arrangement, marital status, college, smoking status, and physical activity. The second section aimed to assess eating habits through questions on frequency of meals (less than three meals/three or more meals per day), skipping breakfast (yes/no), eating with family and friends (rarely/often), daily consumption of water (less than 2 L/2 L or more per day), fruits (yes/no), vegetables (yes/no), dairy (yes/no), proteins (yes/no), and homemade foods (yes/no), as well as the frequency of consuming fast food (rarely/often), fried food (rarely/often), and canned food (rarely/often). The last section included questions on psychological factors that may affect the dietary habits of participants such as “eating when happy”, “eating when stressed”, “eating when sad”, and “eating when bored”. The full version of the questionnaire is available as a Supplementary File (S2).

Anthropometric measurements including height and weight were also assessed. Height (cm) and weight (kg) were measured while wearing light clothes and no shoes, using a mobile stadiometer (SECA 213, Hamburg, Germany) and a digital flat scale (SECA 874, Hamburg, Germany), respectively. Body mass index (BMI) was calculated by dividing the weight in kilograms by the height squared in meters (kg/m^2^). The World Health Organization's (WHO) guidelines for the classification of adults weight according to BMI was used to categorize participants into four groups: underweight (BMI ≤18.5); normal weight (BMI between 18.5 - 24.9), overweight (BMI between 25 - 29.9); and obese (BMI ≥30) [29].

### 2.3 Data analysis

Statistical analyses were performed using the Statistical Package for Social Sciences (version 26.0, SPSS, Chicago, IL, USA) software. Descriptive statistics and frequencies were used to describe sociodemographic characteristics, eating patterns, and psychological factors. Mean and standard deviation (SD) were used to present continuous variables. An eating habits score was calculated based on the number of favorable eating habit responses and it ranged from 0 to 12. Each question about eating habits was scored (0) if the response was not favorable (e.g., eating fried foods often) or (1) if favorable (e.g., drinking 2 L or more of water per day). Differences between eating habits scores were determined using an independent t-test and a one-way ANOVA test Hierarchical multivariate linear regression analysis was used to study the association between eating habits score and sociodemographic characteristics and psychological factors. An independent *t*-test was used to compare mean eating habits score and mean BMI among different psychological factors. *P* values were considered statistically significant at *p* < 0.05.

## 3 Results

### 3.1 Sample characteristics

A total number of 529 students participated in the study. Table 1 shows the sociodemographic and physical characteristics of the participants. The age of participants ranged between 18 and 26 years. The female to male ratio was almost 1:1, with 49.1% males. The majority of the participants were living with their families (57.6%) and were single (96%). Most students were non-smokers (79.2%) and 39.1% of the participants reported not engaging in regular physical activity. Moreover, 37.6% of participants’ BMI were classified collectively as overweight or obese, and 3.8% were classified as underweight.

**Table 1:** Sociodemographic and physical characteristics of study participants (n = 529).

Characteristics	n (%)
Age (years)	
18–21	371 (70.1)
22–26	158 (29.9)
Gender	
Male	260 (49.1)
Female	269 (50.9)
Nationality	
Emirati and GCC	151 (28.5)
Other Arabs	331 (62.6)
Non-Arabs	47 (8.9)
Residential type	
With Family	305 (57.6)
Out of Family	224 (42.4)
Marital status	
Single	508 (96.0)
Married	19 (3.6)
Divorced	2 (0.4)
Smoking	
Yes	110 (20.8)
No	419 (79.2)
Exercising	
No	207 (39.1)
Yes	322 (60.9)
Body Mass Index (kg/m^2^)	
Underweight (≤18.5)	20 (3.8)
Normal weight (18.5–24.9)	310 (58.6)
Overweight (25–29.9)	145 (27.4)
Obesity (≥30)	54 (10.2)

### 3.2 Eating habits

Table 2 presents the eating habits of the study participants. When participants were asked about the number of meals they consumed per day, 35.4% answered that they consumed 1–2 meals per day. Less than half of the participants reported eating breakfast daily (45.4%). While most of them reported usually eating with family and friends (58.2%).

**Table 2:** Eating habits among participants (n = 529).

Eating habits	n (%)
How many meals do you eat per day?	
<3	240 (45.4)
≥3∗	289 (54.6)
Do you have breakfast daily?	
Yes∗	240 (45.4)
No	289 (54.6)
Do you eat with friends and family daily?	
Yes∗	308 (58.2)
No	221 (41.8)
How much water do you drink daily? (L/day)	
<2	440 (83.2)
≥2∗	89 (16.8)
Do you eat fruits daily?	
Yes∗	152 (28.7)
No	377 (71.3)
Do you eat vegetables daily?	
Yes∗	180 (34)
No	349 (66)
Do you consume milk or milk products daily?	
Yes∗	237 (44.8)
No	292 (55.2)
Do you eat meat/chicken/fish daily?	
Yes∗	293 (55.4)
No	236 (44.6)
Do you eat homemade meals daily?	
Yes∗	232 (43.9)
No	297 (56.1)
How often do you eat fast food?	
Rarely∗	246 (46.5)
Often	283 (53.5)
How often do you eat fried food?	
Rarely∗	228 (43.1)
Often	31 (56.9)
How often do you eat canned food?	
Rarely∗	384 (72.8)
Often	145 (27.4)

∗ Favorable eating habits. The eating habits score was calculated based on the sum of favorable responses and it ranged between 0 and 12. A higher score indicates better eating habits.

Moreover, only 16.8% of participants had two liters or more of water intake daily and only 28.7% consumed fruits daily. A higher percentage reported eating vegetables, dairy, and proteins daily (34%, 44.8%, and 55.4%, respectively). More than half of the participants reported not consuming homemade meals daily (56.1%). While the majority consumed fast and fried foods frequently (53.5% and 56.9%, respectively). Most of the participants reported hardly consuming canned food (72.8%).

The total mean eating habit score for the study population was 5.44 (SD = 2.4). As shown in Table 3, non-Arab participants, those living with family, married, non-smokers, and those who engage in physical activity had a higher mean eating habit score (*p <* 0.05).

**Table 3:** Sociodemographic characteristics of study participants with eating habits score (n = 529).

Characteristics	Mean ± SD	*p-value*
Age (years)		
18–21	5.5 (2.4)	0.497
22–26	5.3 (2.5)	
Gender		
Male	5.6 (2.5)	0.266
Female	5.3 (2.4)	
Nationality		
Emirati and GCC	5.1 (2.5)	0.027
Other Arabs	5.5 (2.4)	
Non-Arabs	6.2 (2.5)	
Residential type		
With Family	5.8 (2.6)	<0.001
Out of Family	5.0 (2.2)	
Marital status		
Single	5.4 (2.4)	0.001
Married	6.3 (2.8)	
Smoking		
Yes	4.9 (2.5)	0.012
No	5.6 (2.4)	
Exercising		
No	4.7 (2.2)	<0.001
Yes	5.9 (2.5)	

Mean differences assessed using ANOVA test and independent t-test, significance set at p < 0.0.5.

### 3.3 Psychological factors

Psychological factors affecting the eating practices of the participants are presented in Table 4. Over 80% of the participants reported eating when feeling happy and about 80% ate when feeling bored. Moreover, 56.5% of the participants reported eating when they feel sad. Stress-induced eating was reported by 47.8% of the study participants and 33.6% indicated eating without control.

**Table 4:** Psychological factors affecting eating habits among participants (n = 529).

Psychological factors	n (%)
Eat when happy	
No	86 (16.3)
Yes	443 (83.7)
Eat when sad	
No	230 (43.5)
Yes	299 (56.5)
Eat when stressed	
No	276 (52.2)
Yes	253 (47.8)
Eat when bored	
No	108 (20.4)
Yes	421 (79.6)
Eat uncontrollably	
No	351 (66.4)
Yes	178 (33.6)

Mean eating habits scores were comparable during different psychological aspects, however, those who reported eating uncontrollably has a significantly lower mean eating habits score (5.04 ± 2.3) compared to those who did not (5.64 ± 2.4) (*p* < 0.05) (Table 5). Moreover, the mean BMI for those who ate when stressed and for those who reported eating uncontrollably was significantly higher than those who did not (p = 0.009 and p = 0.006, respectively).

**Table 5:** Comparisons of the eating habits scores and BMI among different psychological factors (n = 529).

Psychological factors	Eating Habit Score Mean (SD)	*p-value*	BMI Mean (SD)	*p-value*
Eat when happy				
No	5.22 (2.5)	0.36	24.41 (4.5)	0.91
Yes	5.48 (2.4)		24.35 (4.2)	
Eat when sad				
No	5.38 (2.4)	0.63	24.02 (4.1)	0.10
Yes	5.48 (2.4)		24.63 (4.4)	
Eat when stressed				
No	5.52 (2.4)	0.42	23.90 (4.0)	0.009
Yes	5.35 (2.5)		24.87 (4.5)	
Eat when bored				
No	5.54 (2.6)	0.61	24.29 (3.8)	0.84
Yes	5.41 (2.4)		24.38 (4.4)	
Eat uncontrollably				
No	5.64 (2.4)	0.007	24.00 (4.2)	0.006
Yes	5.04 (2.3)		25.09 (4.2)	

Mean differences assessed using independent t-test, significance set at p < 0.0.5.

Table 6 shows the association between eating habits score and sociodemographic and psychological variables using hierarchical multivariate linear regression. Nationality, marital status, age, sex, residential type, smoking, and exercising were entered in the first step. In the second step, psychological factors were included ‘eat when happy’, ‘eat when sad’, ‘eat when stressed’, ‘eat when bored’, and ‘eat uncontrollably’. The results from the first step indicated that marital status, residential type, smoking, and physical activity were significantly associated with eating habits score (*p* < 0.05). In the second step, factors associated with eating habits score were marital status, residential type, smoking, exercising, and eating when bored (*p* < 0.05). No multicollinearity was identified between variables and the total model was significant (*p* < 0.001) accounting for 22% of the variance.

**Table 6:** Association of the eating habits scores with different sociodemographic characteristics and psychological factors using hierarchical multiple linear regression (n = 529).

Variables	*Step 1*			*Step 2*		
	B	Beta	*p-value*	B	Beta	*p-value*
Nationality	0.43	0.10	0.07	0.45	0.11	0.07
Marital Status	1.21	0.09	0.03	1.28	0.10	0.02
Age	0.01	0.00	0.97	0.01	0.02	0.96
Sex	0.20	0.04	0.36	0.22	0.05	0.32
Residential type	0.77	-0.15	<0.001	-0.70	-0.14	0.001
Smoking	-0.85	-0.14	0.001	-.77	-2.94	0.003
Exercising	1.31	6.03	<0.001	1.30	6.01	<0.001
Eat when happy				0.31	1.10	0.22
Eat when sad				0.27	1.22	0.38
Eat when stressed				-0.19	-0.87	0.73
Eat when bored				-0.09	-0.34	0.03
Eat uncontrollably				-0.49	-2.17	0.22

The reference group for nationality ‘non-Arab’; for marital status ‘married’; for age is ‘22–26 years'; for sex ‘female’; for residential type ‘out of family’; for all other variables is ‘yes’. P-value assessed using hierarchical multiple linear regression.

## 4 Discussion

Based on the BMI classification of weight status, more than one-third of participants in this study were classified as overweight or obese. This finding was slightly lower than that reported in another study conducted on UOS students (41.7%) [13]. But, higher than the prevalence of overweight and obesity reported in a study conducted on students from three different universities in the UAE (32.9%) [30]. Although BMI does not consider muscle and fat mass [31], but it was used as an indicator for weight status. In this study, over one-third of participants reported not engaging in regular physical activity. Similar findings were reported among female university students in the UAE, where 32.7% of participants reported not exercising during a typical week [32]. Expectedly, the lack of activity is a habitual phenomenon in the Gulf countries [33]. This is a critical situation when physical inactivity is well documented as a key risk factor for many non-communicable diseases including obesity. Knowing that one-third of participants were overweight and obese, warrants additional measures regarding improving physical activity.

In terms of dietary habits, less than half of the participants consumed daily breakfast. This finding is similar to that reported by a Malaysian study in which 43.9% of medical students had breakfast daily [27] as well as 53.3% of university students in Bangladesh [34], while 31.8% of Lebanese university students stated that they had breakfast daily [26]. Skipping breakfast is a common practice among university students, which could influence other eating behaviors such as repeated snacking [35]. A study on high school students in Abu Dhabi found a positive association between consuming breakfast and better academic performance (*p* < 0.001) [36]. The latter study indicated that the main reason for skipping breakfast among students was lack of time, followed by reduced appetite and not feeling hungry [36].

Most participants consumed less than two liters of water per day. Similarly, a research study on university students reported that participants consume a mean of 1.7 L of fluids per day [37]. These values are much lower than the recommended water intake guidelines (2.0 L per day for females and 2.5 L per day for males over 14 years) of the European Food Safety Authority (EFSA) [38]. Considering the importance of having a healthy fluid drinking pattern at this age stage, tailed nutrition intervention to improve fluid intake among university students should be implemented.

Only one-third of the study population consumed fruits and vegetables daily. This finding is comparable to studies from other countries like Syria and Lebanon [26, 39]. These trends were also observed in Bahrain, as 33.5% of males adolescents and 66.5% of females adolescents did not consume fruits daily [40], and in Saudi Arabia were 78% of university students had low consumption of fruit and vegetable [41]. There is mounting evidence of the protective effect of fruits and vegetables against non-communicable diseases (NCDs) [42]. Therefore, the World Health Organization (WHO) recommends consuming a minimum of 400 g per day of fruits and vegetables, which translates to around five servings per day.

Considering the overhead findings, it is important to take advantage of the university years of the participants to improve their practices regarding nutrition and healthy eating habits, create a supportive environment for healthier food choices, engage in regular physical activity, and promote healthy body weight management among students.

Most of the participants reported eating when they are bored, and more than half ate when they were feeling sad. Several studies have described an association between feeling bored and food intake [43, 44]. Moynihan et al (2015) reported a significant, positive relation between state of boredom and calorie, fat, carbohydrate, and protein consumption [43]. However, the literature has been focused on the effect of negative emotions like stress, sadness, loneliness, and boredom, on eating habits and different coping mechanisms including comfort foods related to such emotions. Only quite recently, studies started investigating the effect of positive emotions like happiness on dietary behaviors and eating patterns. In the current study, participants also reported eating when feeling happy. A study on psychology students reported increased food intake during positive emotions among emotional eaters compared to a neutral mood [45]. Moreover, a meta-analysis of thirty-three studies concluded that positive mood is associated with greater food intake compared to neutral mood across healthy participants and patients with eating and weight disorders [46]. The mechanism behind this effect is not fully understood yet, however, both ghrelin and leptin hormones, as well as, several neurotransmitters (serotonin, dopamine, opioids, and GABA) have been closely linked to mood and eating behavior [47]. Findings from human studies support a two-way link between mood and food intake, suggesting that altered mood can lead to altered food choice and intake leading to overeating and obesity [48]. Moreover, certain food types (comfort foods) tend to be preferred under certain psychological situations owing to the impact of these foods on the activity of brain reward centers [49].

Findings revealed that smokers and those who do not engage in physical activity were more likely to have poor dietary behaviors. Similar findings were reported in Canada, as smoking was correlated with the consumption of fewer fruits and vegetables [50]. Similarly, smoking was associated with poor eating behaviors among medical students in Malaysia [27] and university students in China [51]. Moreover, single participants and those not living with their families were also more likely to have poor eating habits in this study. Likewise, Saudi university students who lived alone had a significantly lower eating habits score compared to those who lived with their families [52]. Moore and Harré suggested that adolescents who live away from family tend to make poor food choices and spend less time engaging in physical activity due to the reduced levels of parental supervision and family cohesion [53].

In this study, uncontrollable eating was associated with poor eating habits and higher BMI. Likewise, a 7-year follow-up concluded that disordered eating behavior such as uncontrollable eating was associated with a higher risk for increased BMI in both females and males [53]. Eating when stressed was also associated with higher BMI in the current study. A meta-analysis of longitudinal studies revealed that high-stress level is a risk factor for weight gain and increased adiposity, however, the effect was found very small [54]. Moreover, a systematic review that investigated the effect of stress during university on weight changes among students found inconsistent findings in the literature [55].

The strengths of this research include examining multi-factorial causes affecting eating habits among university students in the UAE. Understanding the circumstances of such multi-factorial causes would help in the development of healthy food promotional activities by university administration, food providers, and health promoters. Results of this study would also to used as a foundation for possible interventional programs regarding healthy eating habits campaigns.

The study can be discussed in light of few limitations due to using a self-reported questionnaire for data collection, which may lead to social desirability bias, over-reporting of healthy habits, or under-reporting of unhealthy habits. The cross-sectional study design which does not allow for causality evaluation. Moreover, the diet-related chronic diseases and eating disorders were not assessed in the current study. Recall bias might be another limitation, as a recall period was not specified in the current study and students were encouraged to report their usual behavior. In addition, the students are not representative of the larger population of young adults in the UAE. This study was conducted pre COVID-19 pandemic, thus it does not reflect the high stressful impact of the pandemic. Future studies are required to determine the risk factors of emotional eating on eating habits during these times of uncertainty.

## 5 Conclusions

Findings in this study showed a trend of poor eating habits among the participants, such as not meeting the recommended intake of fruits, vegetables, water as well as physical activity guidelines. Living alone, smoking, physical inactivity, eating when stressed, and uncontrollable eating were indicators of poor eating behaviors. Accordingly, universities need to design tailored awareness campaigns about weight management and promote active behavior among students. In addition, it is recommended to assess the eating environment in the university and offer mandatory health-related courses at the university. Implementing stress relief programs and weight management programs are also essential irrespective of BMI or weight change.

## Declarations

### Author contribution statement

Leila Cheikh Ismail: Conceived and designed the experiments; Performed the experiments; Analyzed and interpreted the data; Contributed reagents, materials, analysis tools or data; Wrote the paper.

Tareq M. Osaili: Conceived and designed the experiments; Performed the experiments; Contributed reagents, materials, analysis tools or data; Wrote the paper.

Maysm N. Mohamad: Analyzed and interpreted the data; Contributed reagents, materials, analysis tools or data; Wrote the paper.

Mona Hashim: Conceived and designed the experiments; Performed the experiments; Contributed reagents, materials, analysis tools or data; Wrote the paper.

Lily Stojanovska: Analyzed and interpreted the data.

Rameez Al Daour; Dalal Nader; Hanoof Alrayis; Nouf Sultan Alzaabi; Lojain Elbarag; Shaikha Binkhadim; Amjad H. Jarrar: Performed the experiments; Wrote the paper.

Ayesha S. Al Dhaheri: Conceived and designed the experiments; Contributed reagents, materials, analysis tools or data; Wrote the paper.

Hayder Hasan: Conceived and designed the experiments; Analyzed and interpreted the data; Contributed reagents, materials, analysis tools or data; Wrote the paper.

### Funding statement

This research did not receive any specific grant from funding agencies in the public, commercial, or not-for-profit sectors.

### Data availability statement

The data presented in this study are openly available in FigShare at https://doi.org/10.6084/m9.figshare.14632665.v2.

### Declaration of interest's statement

The authors declare no conflict of interest.

### Additional information

No additional information is available for this paper.

## Appendix A Supplementary data

S1_Informed Consent S1_Informed Consent

S2_Study Questionnaire S2_Study Questionnaire

## References

[1] Bank T.W. United Arab Emirates. Availabe Online:. https://data.worldbank.org/country/AE.

[2] Ng S.W., Zaghloul S., Ali H., Harrison G., Yeatts K., El Sadig M., Popkin B.M. (2011). Nutrition transition in the United Arab Emirates. Eur. J. Clin. Nutr..

[3] Al Dhaheri A., Al Ma'awali A., Laleye L., Washi S. (2014). Nutritional knowledge of Emirati traditional foods and body image perceptions among UAE University students. Emir. J. Food Agric..

[4] Hajat C., Shather Z. (2012). Prevalence of metabolic syndrome and prediction of diabetes using IDF versus ATPIII criteria in a Middle East population. Diabetes Res. Clin. Pract..

[5] Henry C.J.K., Lightowler H.J., Al-Hourani H.M. (2004). Physical activity and levels of inactivity in adolescent females ages 11–16 years in the United Arab Emirates. Am. J. Hum. Biol..

[6] Al-Houqani M., Ali R., Hajat C. (2012). Tobacco smoking using Midwakh is an emerging health problem–evidence from a large cross-sectional survey in the United Arab Emirates. PLoS One.

[7] Mehairi A.E., Khouri A.A., Naqbi M.M., Muhairi S.J., Maskari F.A., Nagelkerke N., Shah S.M. (2013). Metabolic syndrome among Emirati adolescents: a school-based study. PLoS One.

[8] Malik M., Razig S.A. (2008). The prevalence of the metabolic syndrome among the multiethnic population of the United Arab Emirates: a report of a national survey. Metab. Syndr. Relat. Disord..

[9] Cheikh Ismail L., Osaili T.M., Mohamad M.N., Al Marzouqi A., Jarrar A.H., Abu Jamous D.O., Magriplis E., Ali H.I., Al Sabbah H., Hasan H. (2020). Eating habits and lifestyle during COVID-19 lockdown in the United Arab Emirates: a cross-sectional study. Nutrients.

[10] Radwan H., Al Kitbi M., Hasan H., Al Hilali M., Abbas N., Hamadeh R., Saif E.R., Naja F. (2021). Indirect health effects of COVID-19: unhealthy lifestyle behaviors during the lockdown in the United Arab Emirates. Int. J. Environ. Res. Publ. Health.

[11] Al Dhaheri A.S., Mohamad M.N., Jarrar A.H., Ohuma E.O., Ismail L.C., Al Meqbaali F.T., Souka U., Shah S.M. (2016). A cross-sectional study of the prevalence of metabolic syndrome among young female Emirati adults. PLoS One.

[12] Sheikh-Ismail L.I., Henry C.J.K., Lightowler H.J., Aldhaheri A.S., Masuadi E., Al Hourani H.M. (2009). Prevalence of overweight and obesity among adult females in the United Arab Emirates. Int. J. Food Sci. Nutr..

[13] Cheikh Ismail L., Hashim M., H Jarrar A., N Mohamad M., T Saleh S., Jawish N., Bekdache M., Albaghli H., Kdsi D., Aldarweesh D. (2019). Knowledge, attitude, and practice on salt and assessment of dietary salt and fat intake among university of Sharjah students. Nutrients.

[14] Musaiger A., Lloyd O., Al-Neyadi S., Bener A. (2003). Lifestyle factors associated with obesity among male university students in the United Arab Emirates. Nutr. Food Sci..

[15] Attlee A., Abu-Qiyas S., Obaid R. (2014). Assessment of nutrition knowledge of a university community in Sharjah, United Arab Emirates. Malaysian J. Nutr..

[16] Kerkadi A. (2003). Evaluation of nutritional status of United Arab Emirates university female students. Emir. J. Agric. Sci..

[17] Ali H.I., Jarrar A.H., Abo-El-Enen M., Al Shamsi M., Al Ashqar H. (2015). Students’ perspectives on promoting healthful food choices from campus vending machines: a qualitative interview study. BMC Publ. Health.

[18] Byrd-Bredbenner C., Johnson M., Quick V.M., Walsh J., Greene G.W., Hoerr S., Colby S.M., Kattelmann K.K., Phillips B.W., Kidd T. (2012). Sweet and salty. An assessment of the snacks and beverages sold in vending machines on US post-secondary institution campuses. Appetite.

[19] Gomathi K.G., Ahmed S., Sreedharan J. (2012). Psychological health of first-year health professional students in a medical university in the United Arab Emirates. Sultan Qaboos Univ. Med. J..

[20] Papier K., Ahmed F., Lee P., Wiseman J. (2015). Stress and dietary behaviour among first-year university students in Australia: sex differences. Nutrition.

[21] Mikolajczyk R.T., El Ansari W., Maxwell A.E. (2009). Food consumption frequency and perceived stress and depressive symptoms among students in three European countries. Nutr. J..

[22] Lee P.C., Ahmed F., Pathirana T., Papier K. (2016). Food and Nutrition Report.

[23] Meule A., Reichenberger J., Blechert J. (2018). Smoking, stress eating, and body weight: the moderating role of perceived stress. Subst. Use Misuse.

[24] Hootman K.C., Guertin K.A., Cassano P.A. (2018). Stress and psychological constructs related to eating behavior are associated with anthropometry and body composition in young adults. Appetite.

[25] Bennett J., Greene G., Schwartz-Barcott D. (2013). Perceptions of emotional eating behavior. A qualitative study of college students. Appetite.

[26] Yahia N., Achkar A., Abdallah A., Rizk S. (2008). Eating habits and obesity among Lebanese university students. Nutr. J..

[27] Ganasegeran K., Al-Dubai S.A., Qureshi A.M., Al-Abed A.-A.A., Rizal A., Aljunid S.M. (2012). Social and psychological factors affecting eating habits among university students in a Malaysian medical school: a cross-sectional study. Nutr. J..

[28] Kagan D.M., Squires R.L. (1984). Compulsive eating, dieting, stress, and hostility among college students. J. Coll. Student Person..

[29] (2000). WHO. *Obesity: Preventing and Managing the Global Epidemic. Report of a WHO Consultation (WHO Technical Report Series 894)*.

[30] Hasan H.A., Najm L., Zaurub S., Jami F., Javadi F., Deeb L.A., Iskandarani A., Radwan H. (2018). Eating disorders and body image concerns as influenced by family and media among university students in Sharjah, UAE. Asia Pac. J. Clin. Nutr..

[31] PiepioraABCDE P., SupersonABCDE M., WitkowskiCD K., MaślińskiCD J., MigasiewiczAE J. (2018).

[32] Matthews J.D. (2018). Health literacy among female university students in the United Arab Emirates. Int. J. Health Promot. Educ..

[33] Mabry R., Reeves M.M., Eakin E.G., Owen N. (2010). Evidence of physical activity participation among men and women in the countries of the Gulf Cooperation Council: a review. Obes. Rev..

[34] Shill K.B., Karmakar P., Kibria M.G., Das A., Rahman M.A., Hossain M.S., Sattar M.M. (2014). Prevalence of iron-deficiency anaemia among university students in Noakhali region, Bangladesh. J. Health Popul. Nutr..

[35] Savige G., MacFarlane A., Ball K., Worsley A., Crawford D. (2007). Snacking behaviours of adolescents and their association with skipping meals. Int. J. Behav. Nutr. Phys. Activ..

[36] Taha Z., Rashed A.S. (2017). The effect of breakfast on academic performance among high school students in Abu Dhabi. Arab J. Nutr. Exercise (AJNE).

[37] Balaghi S., Faramarzi E., Mahdavi R., Ghaemmaghami J. (2011). Fluids intake and beverage consumption pattern among university students. Health Promot. Perspect..

[38] EFSA Panel on Dietetic Products N., Allergies (2010). Scientific opinion on dietary reference values for water. EFSA J..

[39] Bashour H. (2004 2004). Survey of dietary habits of in-school adolescents in Damascus, Syrian Arab Republic. EMHJ-Eastern Mediterranean Health J..

[40] Musaiger A., Bader Z., Al-Roomi K., D'Souza R. (2011). Dietary and lifestyle habits amongst adolescents in Bahrain. Food Nutr. Res..

[41] Al-Otaibi H.H. (2014). The pattern of fruit and vegetable consumption among Saudi university students. Global J. Health Sci..

[42] Wallace T.C., Bailey R.L., Blumberg J.B., Burton-Freeman B., Chen C.y.O., Crowe-White K.M., Drewnowski A., Hooshmand S., Johnson E., Lewis R. (2020). Fruits, vegetables, and health: a comprehensive narrative, umbrella review of the science and recommendations for enhanced public policy to improve intake. Crit. Rev. Food Sci. Nutr..

[43] Moynihan A.B., Van Tilburg W.A., Igou E.R., Wisman A., Donnelly A.E., Mulcaire J.B. (2015). Eaten up by boredom: consuming food to escape awareness of the bored self. Front. Psychol..

[44] Crockett A.C., Myhre S.K., Rokke P.D. (2015). Boredom proneness and emotion regulation predict emotional eating. J. Health Psychol..

[45] Bongers P., Jansen A., Havermans R., Roefs A., Nederkoorn C. (2013). Happy eating. The underestimated role of overeating in a positive mood. Appetite.

[46] Cardi V., Leppanen J., Treasure J. (2015). The effects of negative and positive mood induction on eating behaviour: a meta-analysis of laboratory studies in the healthy population and eating and weight disorders. Neurosci. Biobehav. Rev..

[47] Singh M. (2014). Mood, food, and obesity. Front. Psychol..

[48] Potenza M.N. (2014). Obesity, food, and addiction: emerging neuroscience and clinical and public health implications. Neuropsychopharmacology.

[49] Weltens N., Zhao D., Van Oudenhove L. (2014). Where is the comfort in comfort foods? Mechanisms linking fat signaling, reward, and emotion. Neuro Gastroenterol. Motil..

[50] Barre D.E., Mizier-Barre E., Macintyre P. (2011). Socioeconomic factors and their relation to eating habits in two communities in Nova Scotia, Canada. J. Hunger Environ. Nutr..

[51] Sakamaki R., Toyama K., Amamoto R., Liu C.-J., Shinfuku N. (2005). Nutritional knowledge, food habits and health attitude of Chinese university students –a cross sectional study. Nutr. J..

[52] Alzahrani S.H., Saeedi A.A., Baamer M.K., Shalabi A.F., Alzahrani A.M. (2020). Eating habits among medical students at king abdulaziz university, jeddah, Saudi Arabia. Int. J. Gen. Med..

[53] Moore J., Harré N. (2007). Eating and activity: the importance of family and environment. Health Promot. J. Aust..

[54] Wardle J., Chida Y., Gibson E.L., Whitaker K.L., Steptoe A. (2011). Stress and adiposity: a meta-analysis of longitudinal studies. Obesity.

[55] Haidar S.A., de Vries N.K., Karavetian M., El-Rassi R. (2018). Stress, anxiety, and weight gain among university and college students: a systematic review. J. Acad. Nutr. Diet..

