# Supplementary material for: Psychosocial factors affecting dietary habits of university students: A cross-sectional study
Source: Heliyon. 2022 Jun 22;8(6):e09768. doi: 10.1016/j.heliyon.2022.e09768 (PMC9249847; doi:10.1016/j.heliyon.2022.e09768)
Supplement: S1_Informed Consent [file mmc1.pdf]

جامعة الشارقة
UNIVERSITY OF SHARJAH

كلية العلوم الصحية
COLLEGE OF HEALTH SCIENCES

Department of Clinical Nutrition and Dietetics

Research study: Psychosocial Factors Affecting Dietary Habits of University Students: A Cross-Sectional Study

Informed Consent Form

Respected UOS students:

You are invited to complete a self-administered questionnaire entitled "Psychological Factors Affecting Dietary Habits of University Students: a cross-sectional study". Your weight and height will also be measured.

This research project is conducted by the Department of Clinical Nutrition and Dietetics at University of Sharjah. The study protocol was approved by the Research Ethics Committee at the UOS (REC-18-03-08-01-S).

Your participation in this study is completely voluntary. You may refuse to take part in the research or exit the survey at any time. The researchers will maintain total confidentiality of your responses, and all data will be anonymous without any indication of personal identity. Your responses will help us to examine the effect of social and psychological factors on eating habits and food choices among medical and non-medical students at the University of Sharjah (UOS), UAE.

As a participant in this study, there will be no risks on you when filling out the questionnaire and your weight and height will be measured in a private room in the university clinic. Moreover, no incentives will be provided for taking part in the research.

The survey should take approximately 10-15 minutes to complete.

I have read (or someone has read for me) the information mentioned above. I am a student at UOS. I had the opportunity to ask questions and was satisfied with the answers provided. I agree to participate in this research study.

Name: ___________________ Signature ___________________ Date_______________

Thank you for your participation.

For more information:

If you have any questions or would like a copy of this consent letter, please contact:
Dr. Leila Cheikh Ismail, Department of Clinical Nutrition and Dietetics, College of Health Sciences, University of Sharjah, Sharjah/UAE. Email: lcheikhismail@sharjah.ac.ae

If you have a complaint about this study, kindly contact Dr. Suhail Alamad, Chair of the Research Ethics Committee in University of Sharjah: Salamad@sharjah.ac.ae
