# Supplementary material for: Psychosocial factors affecting dietary habits of university students: A cross-sectional study
Source: Heliyon. 2022 Jun 22;8(6):e09768. doi: 10.1016/j.heliyon.2022.e09768 (PMC9249847; doi:10.1016/j.heliyon.2022.e09768)
Supplement: S2_Study Questionnaire [file mmc2.pdf]

Department of Clinical Nutrition and Dietetics

Research study: Psychosocial Factors Affecting Dietary Habits of University Students: A Cross-Sectional Study

Questionnaire

Measured Anthropometry: Height ______ cm Weight ______ kg

Section A: Socio-demographic Information

1) Age
- ☐ 18-21
- ☐ 22-26

2) Gender
- ☐ Male
- ☐ Female

3) Nationality
- ☐ Emirati and GCC
- ☐ Other Arabs
- ☐ Non-Arabs

4) Marital Status
- ☐ Single
- ☐ Married
- ☐ Divorced
- ☐ Widowed

5) Residence
- ☐ With family
- ☐ Out of family

6) Do you smoke?
- ☐ Yes
- ☐ No

7) Do you exercise (jogging, running, weightlifting, sports training, etc.) in a week?
- ☐ No
- ☐ Yes

Section B: Assessment of Dietary Intake

8) Do you usually eat breakfast?
☐ Yes
☐ No

9) How many meals do you eat per day?
☐ <3
☐ 3+

10) How often do you eat with your friends/family?
☐ Rarely
☐ Often

11) How much water do you drink per day?
☐ Less than 2L
☐ 2L+

12) Do you eat fruits daily?
☐ Yes
☐ No

13) Do you eat vegetables daily?
☐ Yes
☐ No

14) Do you consume milk and milk products daily?
☐ Yes
☐ No

15) Do you eat meat/chicken/fish daily?
☐ Yes
☐ No

16) How often do you eat fast food?
☐ Rarely
☐ Often

17) How often do you eat fried food?
☐ Rarely
☐ Often

18) How often do you eat canned food?
☐ Rarely
☐ Often

19) Do you eat homemade meals daily?
- ☐ Yes
- ☐ No

Section C: Assessment of Psychological Factors

20) Do you:

	**No**	**Yes**
Eat when you are happy		
Eat when you are sad		
Eat when you are stressed		
Eat when you are bored		
Eat uncontrollably		
